# Navigating the complexity of oral peptide delivery: challenges and strategies to enhance oral bioavailability

**DOI:** 10.3389/fddev.2026.1809842

**Published:** 2026-03-24

**Authors:** Noor-Ul-Ain Khalid, Edgardo Rivera-Delgado, Thomas von Erlach

**Affiliations:** Vivtex Corporation, Boston, MA, United States

**Keywords:** gastrointestinal barriers, intestinal permeability, oral bioavailability, oral peptide delivery, permeation enhancers, pharmacokinetics, solid dosage formulations, translational modeling

## Abstract

Peptide therapeutics have emerged as a rapidly expanding drug class capable of modulating highly specific and previously inaccessible molecular targets. Maximizing the clinical and commercial potential of therapeutics, particularly in chronic indications, requires patient-friendly routes of administration with oral delivery often being preferred for its non-invasive nature. Peptides, however, often exhibit unfavorable physicochemical properties for epithelial permeability, paired with high polarity, charge, molecular size, and proteolytic instability. These factors collectively result in low and often insufficient systemic bioavailability. Recent advances in peptide chemistry have shifted the landscape of oral delivery. Structural modifications that enhance potency, metabolic stability, and half-life have enabled therapeutic efficacy even at low single-digit oral bioavailability, as demonstrated by clinically successful and late-stage candidates. Consequently, the central challenge is no longer feasibility, but optimization through designing formulation strategies that are mechanistically aligned with the physicochemical and pharmacokinetic properties of individual peptides. This review examines the key scientific barriers to oral peptide absorption and evaluates emerging strategies to address them, focusing on three interconnected domains: (1) functional excipients to overcome biological barriers; (2) translation of liquid prototypes into robust solid dosage formulations; and (3) integration of *in vitro*, *in vivo*, and computational tools to improve predictive accuracy and accelerate development. Collectively, these approaches outline a framework for data-driven optimization and potential route to de-risk translation of oral peptide therapeutics.

## 1 Introduction

Peptide therapeutics have become an increasingly important drug class on account of their ability to modulate previously inaccessible molecular targets with high specificity and potency (Xiao et al., 2025). Accordingly, a growing number of peptide candidates are advancing through clinical pipelines, across a variety of therapeutic areas, including metabolic, oncologic, autoimmune, inflammatory, and infectious diseases, among others (Baggio and Drucker, 2021; Recio et al., 2017; Vadevoo et al., 2019; Zheng et al., 2025). Maximizing the clinical and commercial potential of therapeutics, particularly in chronic indications, requires patient-friendly routes of administration, with oral delivery often preferred for its non-invasive nature and positive impact on patient adherence and long-term treatment outcomes (Baryakova et al., 2023; Ingersoll and Cohen, 2008). However, peptide therapeutics have historically been limited to parenteral administration, with successful oral delivery remaining a largely unresolved challenge due to unfavorable molecular properties that limit their ability to overcome gastrointestinal barriers (Hamman et al., 2005). Peptides are relatively large, polar and charged, leading to low permeability through the mucus layer and underlining epithelial barrier (Brown et al., 2020; Ensign et al., 2012; Gupta et al., 2013). Additionally, their susceptibility to gastric and intestinal proteases, propensity to aggregate and instability, further hinders oral absorption and complicates the development of robust solid dosage form (Brown et al., 2020; Dening et al., 2025; Zhu et al., 2021). When administered orally, these compounding factors often result in low and insufficient systemic bioavailability (Zhou and Po, 1991; Peng et al., 2023; Zhu et al., 2021).

The field of peptide therapeutics has undergone remarkable advancement in the ability to design highly potent molecules with prolonged half-lives and increased metabolic stability (Xiao et al., 2025). These structural modifications result in favorable pharmacokinetic properties that enable therapeutic efficacy even at low single-digit oral bioavailability, such as those demonstrated by oral semaglutide (Rybelsus®), Merck’s macrocyclic PCSK9 inhibitor MK-0616 (Enlicitide), as well as Protagonist and Janssen’s IL-23 receptor antagonist (Icotrokinra) (Granhall et al., 2019; Johns et al., 2023; Knight et al., 2025). More recent chemistry-driven approaches have further narrowed this gap, as exemplified by the cyclic peptide Luna18 developed by Chugai, which achieves substantially higher oral bioavailability (21%–47%) in preclinical models (Ohta et al., 2023; Tanada et al., 2023). These advances in peptide chemistry have shifted the central challenge of oral peptide delivery from feasibility to optimization, where formulation strategies should be tailored to the physicochemical and pharmacokinetic properties of each peptide, in order to maximize exposure and translational consistency.

**FIGURE 1:**
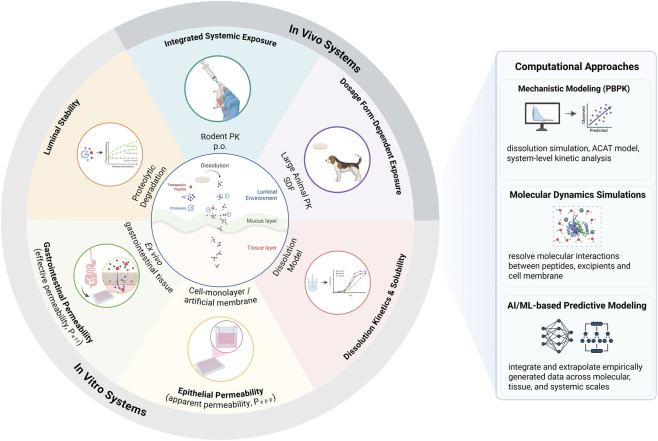
Integrated Experimental and Computational Framework for Evaluating Oral Peptide Absorption. The schematic (center) illustrates key gastrointestinal (GI) barriers and mechanistic processes that influence oral peptide absorption. Experimental models (inner ring) are grouped with their corresponding quantitative readouts, organized according to biological scale, ranging from *in vitro* platforms to integrated *in vivo* pharmacokinetic (PK) studies. Cell monolayers and artificial membrane systems enable assessment of apparent permeability (P_app_), with *ex vivo* gastrointestinal tissue additionally preserving relevant physiological features and cellular architecture. *In vivo* rodent pharmacokinetic (PK) studies provide integrated measures of systemic exposure following oral (p.o.) administration, reflecting the combined impact of luminal stability, permeability, and first-pass metabolism. Large-animal PK studies using solid dosage forms (SDFs) introduce additional formulation-dependent complexity, such as dissolution and solubility considerations. Complementary computational approaches such as mechanistic modeling of dissolution using physiologically based pharmacokinetic (PBPK) frameworks support system-level kinetic analysis, while molecular dynamics simulations help resolve molecular interactions between peptides, excipients and cell membrane. Additionally, AI/ML-based predictive models can integrate and extrapolate empirically generated data across molecular, tissue, and systemic scales. Abbreviations: PE, permeation enhancer; PK, pharmacokinetics; p. o., oral administration; SDF, solid dosage form. Abbreviations: PE, permeation enhancer; PK, pharmacokinetics; p. o., oral administration; SDF, solid dosage form; P_app_, apparent permeability; P_eff_, effective permeability; PBPK, physiologically based pharmacokinetics; ACAT, Advanced Compartmental Absorption and Transit Model. Created in BioRender. Khalid, N (2026) https://BioRender.com/puxkl15.

This review examines the key scientific challenges in oral peptide delivery and highlights emerging strategies to address them, focusing on: (1) functional excipients to overcome biological barriers; (2) solid dosage form design; and (3) experimental and computational tools that support *in vivo* translation (Figure 1).

## 2 The use of functional excipients to overcome biological barriers

### 2.1 Complexity of the gastrointestinal barrier

The gastrointestinal (GI) tract serves as one of the most complex interfaces between the external environment and the human body. Through a series of overlapping barriers, it tightly regulates nutrient uptake while limiting the passage of foreign macromolecules (Schoultz and Keita, 2020). While these features are essential for host protection, they pose significant challenges to oral drug delivery. Peptide-based therapies face multiple inherent challenges due to their unfavorable physicochemical properties: large molecular size, hydrophilicity, structural flexibility and propensity to aggregate (Doak et al., 2014). These properties not only limit passive transport but also restrict paracellular transport through tight junctions that selectively permit the passage of small, neutral molecules (Ménard et al., 2010; Watson et al., 2001). As a result, peptides exhibit inherently low permeability, compounded by the additional upstream barriers they must first overcome.

Before reaching the intestinal epithelium, peptides must surpass several physiological, anatomical and biochemical barriers that significantly reduce the intact fraction available for absorption (Brown et al., 2020; Xu et al., 2021). The acidic gastric environment (pH 1-2), together with proteolytic enzymes such as pepsin, trypsin, and chymotrypsin, promote extensive degradation and shorten residence times (New, 2021; Zhu et al., 2021). Additionally, the mucosal layer represents a major yet often overlooked barrier, acting as a dense, heterogenous matrix that traps and limits the diffusion of hydrophobic or charged peptides (Ensign et al., 2012). Regional variations in mucus composition, thickness, and pH are difficult to reproduce in standard *in vitro* models, the limitations of which are not fully realized in the current formulation design space (Bahari et al., 1982; Brown et al., 2020).

At the epithelial surface, paracellular transport is restrained by tight junctions, whereas transcellular uptake is constrained by efflux transporters and competition with endogenous substrates (Hamman et al., 2005). These transporter-mediated effects remain poorly characterized for both peptides and excipients, adding mechanistic uncertainty to formulation design (Brown et al., 2020; Maher et al., 2016). The intestinal epithelium itself is composed of multiple specialized cell types, each with distinct physiological, metabolic, and transport activity, introducing further complexity to absorption and limiting the translational predictability of preclinical models that lack the complete representation of these cell populations (Brown et al., 2020; Liebing et al., 2025). Additionally, interactions between therapeutic peptides and endogenous luminal peptides or proteins represent an underexplored source of variability that can influence both permeability and stability (Moughan et al., 2014). Together, these barriers impede the successful absorption of peptides and contribute to the low inherent oral bioavailability (<1%) observed for most compounds (Brayden and Maher, 2021; Maher and Brayden, 2021; Zhou and Po, 1991).

### 2.2 Single-excipient formulation strategies

Formulation strategies often rely on functional excipients to address key GI barriers, among which permeation enhancers (PEs) are the most extensively explored tool. Medium-chain fatty acids (MCFAs) such as sodium caprate (C10), bile salts, surfactants, and other amphiphilic molecules aim to transiently increase permeability by modulating tight junctions or disrupting membrane integrity, with some also enhancing membrane fluidity or promoting transcytosis (Maher et al., 2019; Maher et al., 2016). Other excipient classes include protease inhibitors, which reduce enzymatic degradation, and pH modulators, which raise the local pH to stabilize acid-labile peptides and shift ionization states to favor absorption (Brown et al., 2020; Twarog et al., 2019; Welling et al., 2014). Chelating agents, such as EDTA, can also improve paracellular transport by reversibly opening tight junctions through calcium sequestration (Welling et al., 2014).

Although a wide range of excipients target the epithelium, very few directly address the mucus barrier (Brown et al., 2020; Maher et al., 2016). Mucolytics (e.g., N-acetylcysteine), reducing agents, and select surfactants transiently reduce mucus viscosity or disrupt network structure (Boegh and Nielsen, 2015; Peng et al., 2021; Subramanian et al., 2022). Polymer-based materials such as PEGylated or zwitterionic excipients have been explored to improve mucus penetration by reducing adhesive interactions, but these approaches remain largely experimental with minimal translation to oral peptide formulations (Boegh and Nielsen, 2015; Brown et al., 2020). Recent excipient review articles provide only limited depth into the mucus barrier, highlighting that this strategy remains under-characterized and underutilized relative to epithelial PEs (Boegh and Nielsen, 2015; Brown et al., 2020; Zhu et al., 2021).

Active research to identify new functional excipients remains limited, as safety and regulatory hurdles create significant barriers to entry (Baldrick, 2000; Kolter and Guth, 2017; Kozarewicz and Loftsson, 2018). Consequently, only a handful of novel PEs have progressed into commercial use over the last few decades, with sodium salcaprozate (SNAC) in the oral semaglutide tablet (Rybelsus®) being one of the most clinically advanced examples (Aroda et al., 2022; Bohley and Leroux, 2024). SNAC increases membrane fluidity and raises local pH, facilitating transcellular gastric absorption and reducing acid-mediated degradation (Buckley et al., 2018). Its development illustrates the magnitude of investment required to bring a new PE to market, involving nearly 3 decades of extensive toxicological and mechanistic characterization, followed by iterative formulation and regulatory evaluation prior to its incorporation into oral semaglutide (Aroda et al., 2022).

Because of these constraints, formulation development in oral peptide delivery has largely proceeded through iterative optimization within a narrow set of established PEs, rather than broad exploration of new excipient classes (Maher and Brayden, 2021). Although screening studies have demonstrated that additional chemical spaces can modulate epithelial permeability in principle (Lamson et al., 2022), the limited predictive power of simplified *in vitro* models has further reinforced reliance on well-characterized excipients (Brown et al., 2020; Fagerholm et al., 2021).

### 2.3 Functional excipient combinations

Multi-excipient systems can be used to overcome some of these limitations, with several studies showing that combining PEs with complementary co-excipients enhances peptide absorption. For example, Cyprumed reported that trace-metal/reducing-agent systems function as stabilizing or protease-modulating co-excipients, and when combined with a PE, significantly increase intestinal absorption relative to the PE alone (Föger, 2017). In rodents, the GLP-1 peptide, Liraglutide, formulated with the PE sodium dodecyl sulfate (SDS) and a zinc–iron reducing system increased systemic exposure (AUC) by ∼42-fold, compared to SDS alone (Föger, 2017). These systems have been proposed to modulate intestinal protease activity through redox-mediated mechanisms rather than classical competitive inhibition, with similar approaches reported to reduce serine protease activity (Lind et al., 1993). Classical protease inhibitors such as soybean trypsin inhibitor (SBTI), aprotinin, etc., can enhance absorption, but their clinical translation has been limited due to dose requirements, specificity, degradation, and safety considerations (Renukuntla et al., 2013). Trace-metal/reducing systems are proposed to exert localized and transient modulation of protease activity (Föger, 2017), however, validation of their safety profile and comparative performance relative to established excipients such as SNAC, C10, or citrate remains limited, consistent with the broader challenges associated with introducing new functional excipients into oral peptide formulations.

Many pharmaceutical programs combine peptide engineering with formulation design. AstraZeneca developed the orally optimized GLP-1 analogue, MEDI7219, through amino-acid substitutions and bis-lipidation, followed by PE screening to identify the combination of sodium chenodeoxycholate (NaCDC) and propyl gallate (PG) as potential excipients (Pechenov et al., 2021). When formulated as an enteric-coated tablet, MEDI7219 achieved ∼6% oral bioavailability in dogs, a 5-fold increase relative to semaglutide formulated with SNAC (Buckley et al., 2018; Pechenov et al., 2021). Oramed’s insulin formulation (ORMD-0801), relied on the functional combination of SBTI as a peptidase inhibitor, EDTA as a chelator and Tween 80 as a surfactant (Karsdal et al., 2015) but the phase 3 trial was discontinued early due to lack of efficacy (ClinicalTrials.gov, 2023). Eli Lilly reported that co-formulation of an acylated GLP-1/glucagon co-agonist with both C10 as a PE and either soybean trypsin inhibitor or soybean trypsin chymotrypsin inhibitor as protease inhibitor outperformed single-excipient approaches, achieving ∼1% oral bioavailability in minipigs when delivered in enteric-coated capsules (Tran et al., 2022). Tran used a combination of *in situ* experiments in the rat jejunum loop model, alongside vitro protease activity assays using rat derived native intestinal fluids. The addition of a chelator to formulations containing C10 and the peptidase inhibitor did not improve bioavailability (Tran et al., 2022).

### 2.4 Synergistic formulation design

Formulation efforts have largely relied on a small set of well-characterized excipients broadly applied across diverse peptide classes, rarely yielding bioavailability’s beyond the low single-digit range in large animals and humans (Brayden and Maher, 2021; Maher and Brayden, 2021; Niu et al., 2025). The persistence of this apparent “glass-ceiling” suggests that this generalized approach may be insufficient for most peptides. Functional excipients typically address a subset of GI barriers, while others such as mucus diffusion, enzymatic degradation, or efflux transport remain largely unresolved (Maher and Brayden, 2021).

A possible strategy is to identify synergistic combinations capable of modulating multiple barriers at once. These synergies are difficult to predict *a priori*, as mechanism-driven design alone cannot account for the interdependent behavior of excipients within the GI environment (Bardonnet et al., 2025; Karsdal et al., 2015). Their identification requires high-throughput, unbiased screening approaches capable of systematically exploring the vast design space and revealing combinations that would not be apparent using traditional formulation strategies (Folkesson et al., 2020; O’Neil et al., 2016). This concept mirrors directed evolution, a protein engineering strategy in which large combinatorial libraries are generated and screened under selection pressure to reveal functional synergies between mutations that cannot be predicted from the peptide sequence alone (Arnold, 1998).

The discovery of synergistic excipient combinations requires screening systems that reflect the relevant biological complexity. Formulation evaluation has thus heavily relied on *in vivo* studies or *in situ* and *ex vivo* gastrointestinal permeability models, which are time- and resource-intensive and therefore limit the evaluation to a small number of conditions (Tran et al., 2022). This practical limitation is in stark contrast to the potential scale of excipient combinations, which can readily exceed 10^5^ possibilities when considering the number of FDA-listed inactive ingredients (Pottel et al., 2020).

More recently, a high-throughput permeability assay based on intact gastrointestinal tissue has been reported (von Erlach et al., 2020). By using *ex-vivo* tissue, physiological features such as transporter expression, enzymatic activity and the multicellular 3D architecture are retained, more accurately reflecting the interdependent mechanisms that govern permeability (Shi et al., 2024; von Erlach et al., 2020). In a proof-of-concept study using a porcine tissue model, the platform facilitated systematic evaluation of excipient combinations while preserving biological context (von Erlach et al., 2024). Excipient performance was assessed cross-species in rats, with this approach identifying novel formulations yielding up to ∼60% intraduodenal bioavailability for vancomycin, while a distinct combination for semaglutide resulted in ∼10% intraduodenal bioavailability (von Erlach et al., 2024). Translation into oral solid dosage forms (SDF) demonstrated up to ∼5% bioavailability for vancomycin in beagles (von Erlach et al., 2024), exceeding previously reported values of ∼0.3–1.7% for enteric or solution formulations in the same species (Sauter et al., 2020). The identified semaglutide formulations yielded comparable or higher exposure as compared to Rybelsus® (von Erlach et al., 2024). These findings suggest that combinations identified through high-throughput tissue screening may be useful in the development of solid dosage forms.

As experimental throughput expands, active learning may enable iterative prioritization through single- or multi-objective optimization (Bannigan et al., 2023; Hickman et al., 2023). Machine learning models trained on empirical data can be used to establish relationships between chemical properties and experimental outcomes, potentially guiding prioritization of formulation strategies (Reker et al., 2021; Yang et al., 2025). Techniques such as SHapley Additive exPlanations (SHAP) analysis uses collaborative game theory to attribute parts of the prediction response to the features used in a supervised machine learning model (Ponce-Bobadilla et al., 2024) and may further aid in identifying key formulation features. Although robust molecular representations and encoding of formulation features in multi-component mixtures remain a challenge (Bannigan et al., 2023; Chew et al., 2025; Vamathevan et al., 2019). The selection of functional excipients accounts for just one piece of the puzzle. As formulations advance beyond early-stage liquid or solid prototypes, the development of scalable oral dosage forms introduces additional layers of complexity. Excipient compatibility and precise control over release kinetics become critical, placing further constraints on formulation design.

## 3 Solid dosage formulation challenges and considerations

Oral dosage forms are the preferred delivery vehicle due to their convenience, stability, and manufacturing scalability (Hauber et al., 2024; Moges et al., 2024). Key challenges include compartment specific release of the formulation, controlling the release kinetics and the co-localization of the peptide with the functional excipients at the epithelial wall (Maher and Brayden, 2021). If the oral dosage form does not address these challenges, release may occur in the wrong compartment, formulations may dilute or the PEs may separate from the API, all leading to poor translatability.

To overcome these challenges, oral dosages for peptide delivery can be designed as immediate release (IR), delayed release (DR), or controlled release (CR). IR formulations target absorption in the stomach, such as the IR formulation Rybelsus® that uses SNAC to locally raise pH around the tablet, promoting rapid dissolution in the stomach (Buckley et al., 2018). DR peptide delivery systems employ enteric coatings to prevent release in the low pH of the stomach and only release in the small intestine, where pH is higher and the enteric coating can dissolve (Enomoto et al., 2021; Tran et al., 2022). The marketed DR dosage Mycapssa® (oral octreotide) contains sodium caprylate (C8) and stabilizers that are enterically encapsulated to promote intestinal absorption (Tuvia et al., 2014), however oral bioavailability remains low at ∼0.7% in humans (Brayden and Maher, 2021; Tuvia et al., 2012). CR aims to modify the release of the peptide and the PE to prevent asynchronous release and delocalization. To prevent delocalization Syntis Bio has developed GSEL, a mucoadhesive coating that adheres to the duodenal mucosa forming a depot that co-localizes peptide and PEs (Kanelli et al., 2025; Li et al., 2020). To prevent asynchronous release gel forming polymers such as copovidone can be added to oral PE dosages, with composition adjusted to synchronize release (Bapat et al., 2025). Development of these solid dosage strategies relies on extensive experimental evaluation to ensure the chosen strategy chosen can overcome the barriers of the compartment in which it releases.

To shorten development time of new oral dosages, designed experiments can be coupled with machine learning models to predict the performance of new formulation compositions (Bannigan et al., 2023). Computational models to predict solid dosage performance often rely on literature derived data (Bharathi et al., 2024; Galata et al., 2021; Kota et al., 2024). Unlike peptide design (Li et al., 2023), there is currently no centralized and standardized database for solid dosage characterization, but as an increasing number of studies report solid dosage dissolution characterization (Bapat et al., 2025; Buckley et al., 2018; Fagan et al., 2025), this will potentially enable more data-driven modeling approaches. After dosage dissolution, transport of peptide and PE across the GI tract can be modeled with finite element methods to estimate their concentrations at the epithelium (Naranjani et al., 2023; 2026). Integrating mechanistic and data-driven modeling may ultimately indicate physiological conditions and dosage compositions, which are most likely to translate into humans.

## 4 Bridging to human PK: translational barriers

The efficacy of PE formulations is strongly species-dependent, and without optimal experimental designs, promising candidates may fall short in clinical trials. A recent Novo Nordisk study showed that the combined SNAC–C10 formulations increased oral exposure for a GLP-1 analogue and a PCSK9 inhibitor relative to SNAC in beagle dogs, but this effect did not translate to humans (Niu et al., 2025). These discrepancies are thought to be due to the lower gastric pH in humans, relative to dogs, which reduces effective luminal concentration of free C10 in humans (Berg et al., 2022; Harter et al., 1998; Rivera et al., 2023). Further work by Bardonnet demonstrated that controlling the gastric pH in dogs to reflect that of humans, facilitated the identification of the pH modifying excipient meglumine which increases peptide bioavailability of C10 formulations both in dogs and in humans (Bardonnet et al., 2025). Nonetheless, the extent of enhancement relative to SNAC was higher in dogs than in humans, where C10 with meglumine performed similarly to SNAC. These discrepancies, even after careful control of pH are believed to be caused by differences in gastrointestinal physiology between species, such as pore size and frequency of tight junctions (He et al., 1998; Martinez et al., 2021; Twarog et al., 2020).

A multitude of interspecies differences contributes to the lack of translatability between *in vivo* models and human outcomes. Differences in tolerable excipient doses between species further complicate translation considerations (Gad et al., 2016). Dogs can generally tolerate clinically equivalent doses of excipients, whereas non-human primates (NHP) models often require lower doses due to GI sensitivity (Gad et al., 2016). This downscaling between species limits the achievable luminal concentration and thereby lowers the functional capacity of the active excipient (Berg et al., 2022; Maher and Brayden, 2021). Most rodent studies are conducted using liquid formulations, whereas dogs and humans often receive solid oral dosage forms, introducing differences in dissolution kinetics, local excipient concentration, and absorption windows (Cao et al., 2006; Hatton et al., 2015; Henze et al., 2019). Minipigs, despite physiological similarities to humans, frequently display disproportionately high intestinal motility or mucus thickness, leading to over- or underestimation of peptide absorption (Hatton et al., 2015; Henze et al., 2019). Addressing these differences between the model species and humans can increase the number of candidates that translate into humans.

Physics-based molecular modeling can provide valuable mechanistic insight for the formulations approaches that are currently utilized. All-atom molecular dynamics (MD) simulations were used to elucidate the absorption mechanisms of semaglutide in the presence of SNAC (Colston et al., 2025). Similarly, all-atom and coarse-grained simulations have examined peptide-to-excipient and peptide-to-environment interactions, showing how MCFAs, bile salts and other PEs influence peptide aggregation, spatial distribution of excipients in micelles and PE interactions with membranes (Hossain et al., 2019; 2022; 2023; Kneiszl et al., 2022). Physiologically based pharmacokinetic (PBPK) frameworks, such as the ACAT model, provide a mechanistic bridge between simulation outputs and *in vivo* performance by integrating species-specific physiological parameters, formulation-dependent release kinetics, and absorption mechanisms to better predict human exposure (Agoram et al., 2001). Although a common approach for small molecule PBPK modeling (Habiballah and Reisfeld, 2023), it remains underexplored in oral peptide development pipelines. Only recently has such modeling been coupled with MD simulations to help elucidate the oral absorption behavior of semaglutide (Agarwal and Haworth, 2025).

## 5 Conclusions and perspective

Despite decades of research, oral peptide delivery remains a largely unresolved challenge. The physiochemical properties of peptides make them particularly difficult to absorb into systemic circulation, although advancements in peptide chemistry aim to lower the threshold for viable oral bioavailability through increasing potency and extending half-lives. These improvements are reflected in the recent uptake of clinically successful or near-approval oral peptide therapies, that achieve meaningful efficacy even at low single-digit oral bioavailability. The field of oral peptide delivery is entering a new era of development, where the central question is no longer whether oral delivery is viable, but rather how can it be effectively optimized.

Although formulation strategies have shifted away from single-component enhancers, towards rationally designed multi-excipient systems, improvements in oral bioavailability have remained incremental. Functional and rational excipient selection leads to additive effects at best. Evaluating a broader design space by exploring synergistic excipient combinations could allow for the identification of new excipient combinations specifically tailored to each peptide compound and physiochemical and pharmacokinetic properties. This approach requires high-throughput screening assays that model biological complexity, as well as complementary computational tools that can prioritize excipient combinations and elucidate their mechanisms.

Additionally, viable oral formulations depend on the ability to translate liquid prototypes into clinically and commercially viable solid dosage formulations that maintain sufficient oral exposure. This transition currently relies on empirical evaluation in large-animal models, leading to significant costs as well as logistical and resource bottlenecks. Potential acceleration of solid dosage optimization could be achieved through integrating *in vitro* dissolution and permeability evaluation with computational models that account for interspecies GI dynamics. Similarly, enhancing translational confidence will likely require computational augmentation of existing large animal models to incorporate human-specific physiology as well as excipient–peptide interactions under clinically relevant conditions. Collectively, these approaches outline a framework for data-driven optimization and potential route to de-risk translation of oral peptide therapeutics.
